# A Rare Case of Advanced Synchronous Primary Ovarian and Cervical Cancer

**DOI:** 10.7759/cureus.24876

**Published:** 2022-05-10

**Authors:** Mahmoud Abdelsamia, Osama Mosalem, Venumadhavi Gogineni, Keerthi Gullapalli, Eghosa Olomu

**Affiliations:** 1 Internal Medicine, Michigan State University/Sparrow Hospital, Lansing, USA; 2 Internal Medicine, Michigan State University, East Lansing, USA; 3 Radiology, Michigan State University/Sparrow Hospital, Lansing, USA

**Keywords:** cancer immunotherapy, cervical cancer screening, serous ovarian adenocarcinoma, cervical adenosquamos carcinoma, synchronous malignancies

## Abstract

Synchronous gynecological malignancies are rarely encountered, with a growing tide to recognize these primary tumors. However, the most observed synchronous gynecological malignancies remain ovarian and endometrial cancer. This case report presents a 35-year-old female who presented to her gynecologist with lower back pain and dysuria. Transvaginal ultrasound demonstrated a 3-4 cm irregular mass in the cervix and lower uterine segment. Pathology from cold knife conization and endometrial curetting showed serous adenocarcinoma with probable lymphovascular invasion. The patient underwent a positron emission tomography scan that demonstrated an abnormal-appearing cervix, a small number of ascites, peritoneal carcinomatosis, and abnormal left adnexa. Eighteen days later, the patient underwent exploratory laparotomy with total abdominal hysterectomy, bilateral salpingo-oophorectomy, omentectomy, lymphadenectomy, and bowel resection. Surgical histopathological findings confirmed the presence of two primary malignant tumors, namely, cervical adenosquamous carcinoma and bilateral ovarian high-grade serous carcinoma, with extensive metastatic lesions. Although synchronous ovarian and cervical cancer is rarely encountered, patients might benefit from early identification and subsequent debulking surgery with curative intent, as well as adding an immune checkpoint inhibitor in case it is positive on checking as it might improve long-term outcomes.

## Introduction

Synchronous gynecological malignancies are rarely encountered, with an estimated incidence of approximately 1-6% [1]. Primary endometrial and ovarian cancers comprise the vast majority of the observed synchronous tumors [1]. It is crucial to distinguish the origin of these neoplasms, whether primary or metastatic, as early recognition of low-grade and low-stage genital synchronous malignancies usually carries an overall favorable prognosis as opposed to advanced metastatic tumors, as observed in our case.

We present a case of two aggressive Mullerian carcinomas with glandular differentiation, namely, stage IIIA metastatic high-grade serous carcinoma of bilateral ovarian origin and stage IV metastatic cervical adenosquamous carcinoma.

## Case presentation

**Figure 1:**
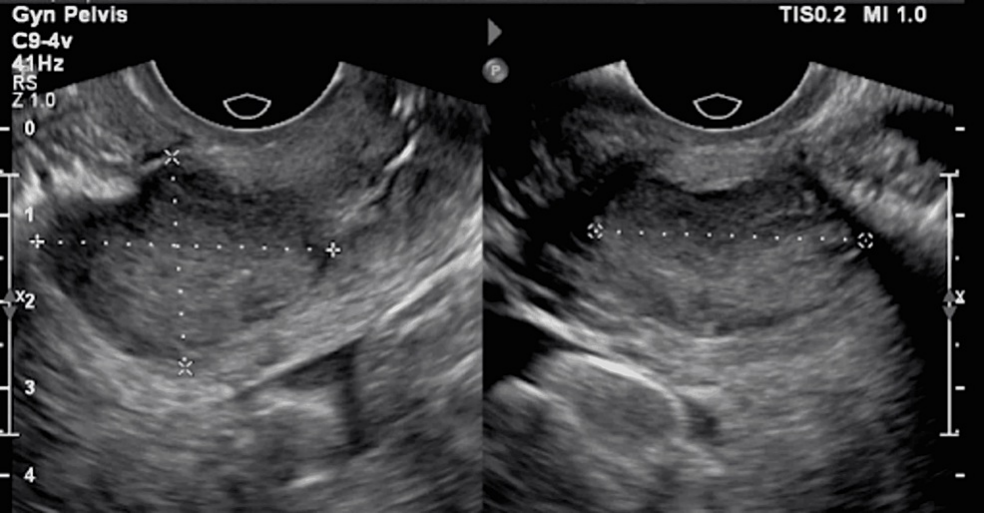
TVUS showing an irregular solid mass in the lower uterine segment measuring 3.5 × 2.45 × 3 cm. TVUS: transvaginal ultrasound

A 35-year-old female with a history of human papillomavirus (HPV) 16 infection, which led to her initial diagnosis of cervical adenocarcinoma, and a family history significant only for breast cancer in her maternal grandmother presented to her gynecologist’s office with lower back pain, bilateral hip pain, dysuria, and suprapubic pain that started three months before presentation. Physical examination was significant for uterine/cervical mass. Cancer antigen-125 (CA-125) was found to be elevated at 409 U/mL. Transvaginal ultrasound (TVUS) demonstrated a solid 3.5 × 2.45 × 3 cm irregular mass in the lower uterine segment, possibly extending up from the cervix (Figure 1). The pathology from cold knife conization showed serous adenocarcinoma extending to the deep resection margin with probable lymphovascular invasion. In addition, it revealed a high-grade squamous intraepithelial lesion (severe squamous dysplasia/CIN 3). Endometrial curettage showed keratinizing poorly differentiated squamous cell carcinoma.

**Figure 2:**
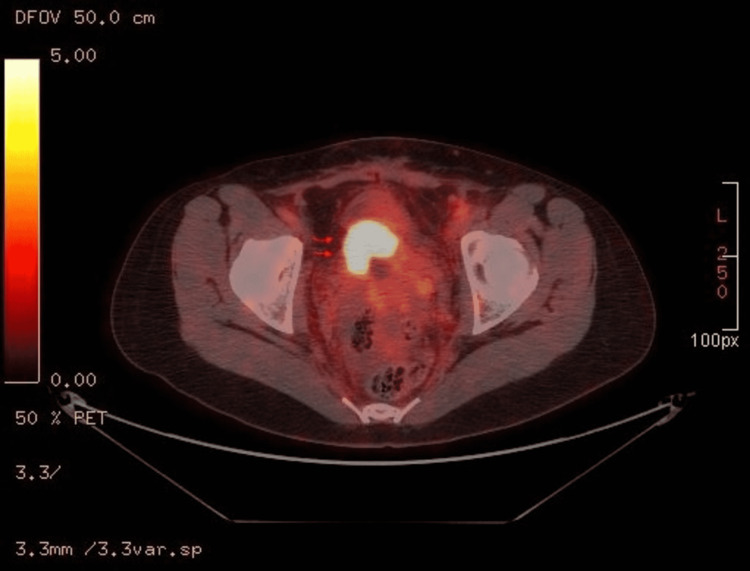
PET-CT showing enlarged intensely FDG-avid cervical mass consistent with cervical carcinoma (SUV of 55.5). PET-CT: positron emission tomography-computed tomography; FDG: fluorodeoxyglucose; SUV: standardized uptake value

The patient subsequently underwent positron emission tomography (PET) that demonstrated abnormal cervix, mild ascites, peritoneal carcinomatosis, and abnormal left adnexa (Figure 2). The patient underwent exploratory laparotomy with total abdominal hysterectomy, bilateral salpingo-oophorectomy, omentectomy, lymphadenectomy, and bowel resection. Surgical histopathological results confirmed the presence of two primary malignant tumors, namely, poorly differentiated cervical adenosquamous carcinoma with a pathological stage of pT1b, pN1, pM1 and bilateral ovarian high-grade serous carcinoma with extensive metastatic lesions involving bilateral fallopian tubes, omentum, peritoneum, abdominal wall, and uterus with pathological stage pT3c, pN1b, pM1b.

Subsequent extensive genetic testing revealed that human epidermal growth factor receptor 2 (*HER2/neu*) was positive while breast cancer genes (*BRCA 1/2*) were negative. Accordingly, the patient was scheduled for six cycles of chemotherapy with carboplatin and docetaxel (docetaxel substituted due to paclitaxel reaction) along with anti-HER2 therapy maintenance. The patient completed four cycles of trastuzumab and continue on pertuzumab.

Outcome/Follow-up

**Figure 3:**
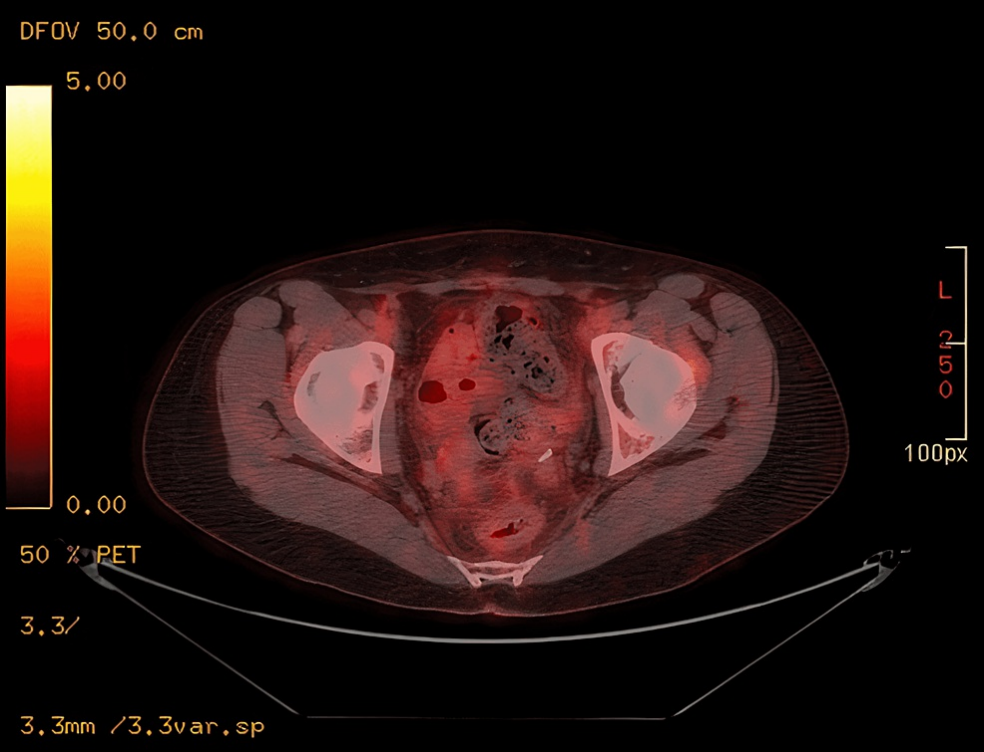
Follow-up PET-CT showing resolution of FDG-avid mass in the pelvis status post-hysterectomy. PET-CT: positron emission tomography-computed tomography; FDG: fluorodeoxyglucose

The patient finished her chemotherapy course and four cycles of pertuzumab and was maintained on trastuzumab. After finishing the chemotherapy, a PET scan showed evidence of complete remission (Figure 3).

The patient reported increased right groin pain and tenderness over a couple of months associated with a rise in CA-125 from 39 U/mL to 87 U/mL. Inguinal ultrasound showed three enlarged right inguinal nodes and one enlarged left inguinal node, which was confirmed by subsequent CT of the chest, abdomen, and pelvis with enlarged lymph nodes in the right inguinal area measuring up to 1 cm in the short axis. A needle biopsy of the right inguinal node revealed metastatic carcinoma, morphologically and immunophenotypically consistent with the patient’s known cervical adenosquamous carcinoma. Subsequent follow-up with PET demonstrated the development of hypermetabolic left supraclavicular, internal mammary, retroperitoneal, pelvic, and inguinal lymph nodes suspicious for recurrent disease. Therefore, trastuzumab was discontinued, and she was initiated on a combination of cisplatin, docetaxel, and bevacizumab (docetaxel substituted due to paclitaxel reaction), and the patient finished six cycles of treatment.

The patient underwent testing for immune checkpoint inhibitors, and programmed death ligand-1 was positive (2%). Follow-up PET showed disease progression; hence, she was started on targeted immunotherapy with pembrolizumab. Unfortunately, subsequent CT of the chest, abdomen, and pelvis demonstrated new and enlarging nodes within the central mesentery, retroperitoneum, and pelvis, suspicious for metastatic disease. There was also a new 8 mm nodule in the right middle lung lobe and multiple mildly/borderline enlarged lymph nodes in the subcarinal, right paratracheal, supraclavicular, and left internal mammary regions. Given the patient’s progression on pembrolizumab, the case was discussed in the tumor board that recommended palliative cytotoxic therapy. Subsequently, the patient initiated a combination of topotecan, docetaxel, and bevacizumab.

Shortly after, due to the patient’s symptoms, her progressing disease, and poor performance status, she opted for hospice care and eventually passed away.

## Discussion

Cervical cancer is the fifth most deadly cancer in women worldwide [2]. Synchronous genital tract malignancies are rare and comprise 0.63% of all genital malignancies. In those with primary synchronous gynecological cancers, primary ovarian with co-existing endometrial cancer is the most observed in 40% of the cases, surprisingly with a favorable prognosis [1]. There are very few cases reported in the literature that present with synchronous ovarian and cervical cancer, as in our case. In a clinical analysis performed by Tong et al., only 20 patients suffered from synchronous gynecological tumors, and only two were noted to have synchronous ovarian and cervical cancer [1]. The etiology remains unclear. However, the most recognized hypothesis is that embryologically similar tissues may develop synchronous neoplasms when simultaneously exposed to hormonal effects or carcinogens [1,3].

The most important question when we face such cases remains whether the two lesions are truly primary in origin or represent metastatic disease. The answer to this question alters the treatment approach and ultimately the overall prognosis, which can be achieved when the observed lesions exhibit different histopathological subtypes or areas of normal parenchyma can be distinguished between the two lesions.

The incidence of cervical cancer has decreased due to organized screening programs. In contrast, the absolute incidence and the relative proportion of adenocarcinoma or adenosquamous carcinoma have increased [4,5]. The hypothesized reason is that screening is less effective against adenocarcinoma than squamous carcinoma of the cervix [6,7]. According to a large observational study performed by Castanon et al., cervical screening by cytology often fails to prevent adenocarcinoma of the cervix. However, it can lead to early diagnosis and down-staging [7]. The study concluded that even though cytology may lack sensitivity for the detection of adenocarcinoma precursors, it is sensitive for detecting early-stage adenocarcinoma. Moreover, the authors observed that the odds of developing stage 1B or worse cancers were reduced in women with up-to-date screening. These results align with the findings of available literature [8,9].

The significance of adenocarcinoma histology as opposed to adenosquamous carcinoma remains inconclusive. The inclusion of adenosquamous carcinoma as a subtype of adenocarcinoma has downplayed its importance in many clinical-pathological analyses that have examined the prognosis and outcomes of patients with cervical adenocarcinoma. Chen et al. reported that patients with adenosquamous histology had a higher percentage of poorly differentiated tumors than patients with adenocarcinoma; these findings correlate with similar findings of other research groups. Wang et al. demonstrated a higher tumor grade and more vascular invasion in adenosquamous carcinoma than in adenocarcinoma [11]. Farley et al. [12] identified 66% grade 3 tumors in patients with adenosquamous carcinoma and 26% in those with adenocarcinoma. In light of these findings, Chen et al. concluded that there were no significant differences in age, International Federation of Gynecology and Obstetrics stage, gravidity, or treatment modality between adenocarcinoma or adenosquamous carcinoma. Based on these findings, adenosquamous carcinoma could be categorized as a subtype of adenocarcinoma [10]

In patients with recurrent, metastatic, and advanced metastatic cancer who are not surgical candidates, platinum-based combination chemotherapy plus bevacizumab has been suggested as first-line therapy based on the results of the Gynecologic Oncology Group 240 trial [13], which revealed an improvement of 3.7 months in median overall survival.

Immunotherapy has been proposed as a treatment modality for advanced cervical cancer, with multiple ongoing trials studying the potential benefit of immunotherapy. The promising results of the open-label, phase II, multi-cohort KEYNOTE-158 trial, which investigated the clinical utility of pembrolizumab in advanced cervical cancer, led to the Food and Drug Administration’s approval of pembrolizumab for the treatment of relapsed or metastatic cervical cancer after frontline chemotherapy treatment, but only for patients whose tumors express programmed death-ligand 1 [14].

## Conclusions

Although synchronous ovarian and cervical cancer is rarely encountered, these patients might benefit from early identification and subsequent debulking surgery with curative intent. Moreover, available published data for the type of treatment of cervical adenocarcinoma focus on the necessity of including targeted monoclonal antibodies, especially in advanced cases, as in our case, as well as adding immune checkpoint inhibitors in positive cases on checking as it might improve long-term outcomes.
